# Active Disturbance Rejection Control Design Using the Optimization Algorithm for a Hydraulic Quadruped Robot

**DOI:** 10.1155/2021/6683584

**Published:** 2021-03-15

**Authors:** Yuqi Fan, Junpeng Shao, Guitao Sun, Xuan Shao

**Affiliations:** Key Laboratory of Advanced Manufacturing and Intelligent Technology, Ministry of Education, School of Mechanical and Power Engineering, Harbin University of Science and Technology, Harbin 150080, China

## Abstract

To increase the robustness and control precision of a hydraulic quadruped robot and simultaneously enhance the dynamic and steady characteristic of the hydraulic system, an active disturbance rejection controller (ADRC) tuned using the Lévy-flight beetle antennae search algorithm (LBAS) was proposed. Moreover, the designed controller was used in the hydraulic quadruped robot to enhance the control performance and restrain the disturbances. The use of the Lévy-flight trajectory in the advanced algorithm can help increase the search speed and iteration accuracy. In the LBAS-ADRC, the parameter tuning method is adopted to develop the active disturbance rejection controller enhanced using the beetle antennae search algorithm. When implemented in the hydraulic quadruped robot, the LBAS-ADRC can ensure satisfactory dynamic characteristics and stability in the presence of external interference. In particular, in the proposed method, the ADRC parameter search problem is transformed to a sixteen-dimensional search problem, the solution of which is identified using the Lévy-flight beetle antennae search algorithm. Moreover, three different algorithms are implemented in the active disturbance rejection controller tuning problem to demonstrate the control performance of the proposed controller. The analysis results show that the proposed controller can achieve a small amplitude overshoot under complex and changeable environments.

## 1. Introduction

Hydraulic quadruped robots have attracted considerable research attention due to their large loading capacity and mobility in complex environments in which robots must execute different tasks [1–3]. Hydraulic quadruped robots are mainly driven by hydraulic servosystems [4, 5]. The active-movement joints of the four legs in hydraulic quadruped robots are controlled by a large integrated valve-controlled hydraulic cylinder. When hydraulic quadruped robots are in motion, a bilateral feedback force exists between the legend and collision object [6]. Consequently, hydraulic driving units must exhibit large control precision and the ability of bending and absorbing the shock of the hydraulic systems to protect the physical and mechanical parts of the robot from impact and damage. Due to the uncertainty in the oil temperature, unknown external loading, and high stiffness variation, hydraulic control systems are nonlinear. Thus, the synchronization and tracking performances of the control systems must be determined to formulate the ideal control strategy and develop an optimized controller [7–12].

The active disturbance rejection controller (ADRC) was proposed by Han Jingqing. The ADRC not only inherits the advantages of the PID but also is less dependent on precise system models. However, there are many parameters in the ADRC controller. Considering the parameter variation of the ADRC controller, the controlled system cannot achieve the perfect performance, which will cause some bad effects for the robot safety campaign [13–17].

The ADRC has been used in many fields, such as MIMO nonlinear systems [18], the hypersonic vehicle [19], temperature control [20], the nonlinear single-input-single-output system [21], permanent-magnet synchronous motors [22], the single-link flexible joint manipulator [23], and coal-fired power plant [24]. It is important to know the main controller parameters [25–29]. Researches for ADRC tuning methods based on metaheuristic algorithms have been given by scientists [30–32].

Jiang and Li in 2017 introduced a metaheuristic optimization algorithm called the beetle antennae search algorithm (BAS) which was inspired by navigating and foraging of beetles in nature [33]. BAS has been used in many fields [34–42]. BAS also has many algorithm variants that are used in some engineering areas [43–45]. The literature [45] proposed the Lévy-flight beetle antennae search algorithm (LBAS) to improve the searching ability while simultaneously tuning the PID parameters in hydraulic systems.

In this paper, position-based ADRC control in hydraulic systems was the focus of research, and LBAS was applied in the ADRC controller to find optimal parameters for leg control of hydraulic quadruped robots. Three major contributions in this paper are summarized as follows:

1. The composition and the principle of ADRC are introduced. Then, this paper defines the tuning parameters that ADRC needs to set, and tuning methods are analyzed. This paper also gives the final mathematics model of the hydraulic system.
2. The application problem of LBAS is studied in parameter tuning optimization of the ADRC controller. According to algorithm characteristics, a third-order ADRC controller is designed based on LBAS. And ADRC parameters tuning problem was converted into the sixteen-dimensional problem. All results show that the ADRC with the tuning parameters optimized by LBAS owns a better anti-interference performance.
3. This paper takes the ADRC controller of the hydraulic quadruped robot as an example, which displays that the improved ADRC has a better control performance in the hydraulic quadruped robot.

## 2. Hydraulic Quadruped Robot Model

**Figure 1:**
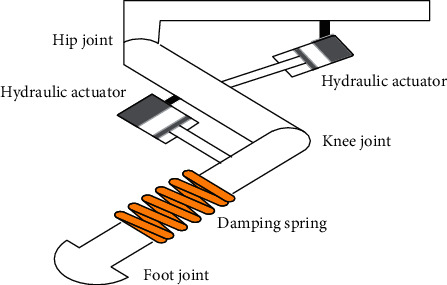
Single leg mechanical structure of the hydraulic quadruped robot.

The hydraulic quadruped robot, which is a mobile platform, mainly includes the trunk and four legs, and each leg has three active joints including the rolling hip joint, the pitching hip joint, and the pitching knee joint. All joints are controlled by electrohydraulic actuators. The hydraulic quadruped robot moving is mainly driven by the leg-swing which is controlled by the hydraulic control systems. The hydraulic quadruped robot platform integrates the robot body, the electrohydraulic servoactuator, the lower-upper computer controller, the signal conditioning circuit, the communication module, the power supply system, and so on [46–54]. The one-leg mechanical structure of the hydraulic quadruped robot is shown in Figure 1.

**Figure 2:**
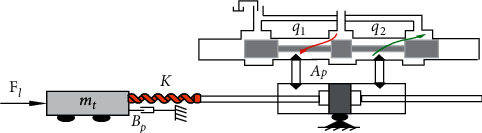
Simplified physical model of the hydraulic system.

The single leg mechanical structure in Figure 1 includes the hip joint, hydraulic actuators, the knee joint, the damping spring, and the foot joint. From Figure 1, we can see that two hydraulic actuators drive the robot leg work. The hydraulic actuator transmission is stable and reliable and is suitable for the working environment with higher transmission requirements. The hydraulic actuator not only has high adjustment precision and a fast response speed but also can realize the high precision control. The designed ADRC will control the hydraulic actuator. The leg moving is mainly controlled by a hydraulic actuator, so control performances of hydraulic actuators are important. To show the working principle of hydraulic control systems, a simplified physical model of the hydraulic servosystem is shown in Figure 2. From Figure 2, we can derive the model of the valve-controlled hydraulic cylinder and then get the final mathematics model. In Figure 2, *A*_*p*_ (m^2^) is the effective area of the piston, *m*_*t*_ (kg) is the piston mass, *B*_*p*_ (N/(m/s)) means the viscous damping coefficient, *K* (N/m)is the loading spring stiffness, and *F*_l_ is some external arbitrary loading; *q*_1_ is the inlet-oil flow, and *q*_2_ is the return-oil flow.

**Figure 3:**
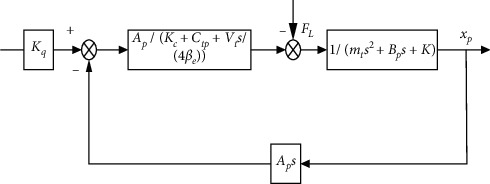
Block diagram of the hydraulic system.

Figure 3 is the block diagram of hydraulic cylinder displacement obtained from the system loading. The final mathematics model can be derived by

(1) $$\begin{array}{c} G\left(s\right)=\frac{\left(K_{q}|/|A_{p}\right)x_{v}-\left(K_{ce}|/|A_{p}^{2}\right)\left(1+\left(V_{t}|/|4\beta_{e}K_{ce}\right)s\right)F_{L}}{\left(m_{t}V_{t}|/|4\beta_{e}A_{p}^{2}\right)s^{3}+\left(\left(m_{t}K_{ce}|/|A_{p}^{2}\right)+\left(B_{p}V_{t}|/|4\beta_{e}A_{p}^{2}\right)\right)s^{2}+\left(1+\left(B_{p}K_{ce}|/|A_{p}^{2}\right)+\left(KV_{t}|/|4\beta_{e}A_{p}^{2}\right)\right)s+\left(KK_{ce}|/|A_{p}^{2}\right)}. \end{array}$$

## 3. Active Disturbance Rejection Control

The ADRC does not depend on the precise system model and can directly estimate the system states and total system disturbances by using the input-output information of the controlled objects, thereby ensuring a high performance in various environments. The ADRC controller is composed of three parts, including the tracking differentiator (TD), nonlinear state error feedback control law (NLSEF), and extended state observer (ESO). The tracking differentiator can promptly track the input signal and provide the output signal. The ADRC uses the tracking characteristics of the extracted differential signal to formulate the signal transition arrangement between the input and differential signals. When the signal response changes abruptly, the TD can promptly provide the smooth signal as the input signal to the controlled system, thereby ensuring that a large overshoot due to the mutations is not incurred and the system stability is enhanced. Furthermore, the TD can ensure high filter performances when the controlled systems are subjected to disturbances. The ESO can identify the signal state variables and assess the external disturbances to realize the system compensation. The disturbance between the plant and model is changed to a new system expansion state by the ESO. The NLSEF determines the control amount by applying the linear combination of the errors. The nonlinear error feedback control law can be used to integrate the error and differential signals generated by the ESO and TD, respectively. In this paper, the controlled model is a three-order system; therefore, the ADRC is constructed for a three-order physical plant in this paper.

TD can smooth the sharp change and arrange the transition procedure in systems. For the simple and typical TD form, output signals can realize the transition arrangement of the input signal and reasonable differential extraction of the input signal [55]. The differential equation of the fast tracking differentiator can be given as follows:

(2) $$\begin{array}{c} \left\{\begin{array}{c} {\dot{x}}_{1}=x_{2},\\ {\dot{x}}_{2}=-r_{1}\text{sign}\left(x_{1}-R\left(t\right)+\frac{x_{2}\left|x_{2}\right|}{2r_{1}}\right), \end{array}\right) \end{array}$$

where *r*_1_ is an adjustable TD parameter and sign() means the standard sign function. *x*_1_ and *x*_2_ are output signals of TD. When *x*_1_ is sufficiently close to the input signal, the input signal can be seen as the approximated differential of the input signal *R*(*t*).

In this paper, the controlled model is a three-order system; therefore, the ADRC is constructed for a three-order physical plant in this paper. The state variable expression is as follows, where *h* = 1:

(3) $$\begin{array}{c} \left\{\begin{array}{c} {\dot{x}}_{1}=x_{2},\\ \vdots \\ {\dot{x}}_{n-1}=x_{n},\\ {\dot{x}}_{n}=r_{1}^{n}f\left(x_{1}-R\left(t\right),\frac{x_{2}}{r_{1}},\ldots ,\frac{x_{n}}{r_{1}^{n-1}}\right). \end{array}\right) \end{array}$$

The discrete TD mathematical model is given as follows:

(4) $$\begin{array}{c} \left\{\begin{array}{c} x_{1}\left(k+1\right)=x_{1}\left(k\right)+x_{2}\left(k\right),\\ x_{2}\left(k+1\right)=x_{2}\left(k\right)+\text{fst}\left(\cdot \right),\\ x_{3}\left(k+1\right)=\text{fst}\left(\cdot \right), \end{array}\right) \end{array}$$

where *x*_1_(*k*), *x*_2_(*k*)_,_ and *x*_3_(*k*) are output signals of TD and *k* is the sampling number.

This paper takes the simplest second-order discrete system as an example to derive the control function fst. First, ADRC uses the control sequence *u*(0),…, *u*(*k*) to reach the initial point expression of the origin [*x*_1_(0), *x*_2_(0)]^T^. Then, the control quantity is the optimal control quantity of the initial point, so this expression is the optimal control synthesis function [55]. The system solution expression of the initial value [*x*_1_(0), *x*_2_(0)]^T^ can be given as follows:

(5) $$\begin{array}{c} \left[\begin{array}{c} x_{1}\left(k\right)\\ x_{2}\left(k\right) \end{array}\right]=\left[\begin{array}{cc} 1 & kh\\ 0 & 1 \end{array}\right]\left[\begin{array}{c} x_{1}\left(0\right)\\ x_{2}\left(0\right) \end{array}\right]+\cdots +\left[\begin{array}{c} 0\\ h \end{array}\right]u\left(k-1\right). \end{array}$$

For a given initial point [*x*_1_(0), *x*_2_(0)]^T^, if *k* control quantities can make the left side of the above formula equal zero, the initial point expression of the origin can be obtained in *k* steps.

(6) $$\begin{array}{c} \left[\begin{array}{c} x_{1}\left(0\right)\\ x_{2}\left(0\right) \end{array}\right]=\left[\begin{array}{c} kh^{2}\\ -h \end{array}\right]u\left(k-1\right)+\cdots +\left[\begin{array}{c} h^{2}\\ -h \end{array}\right]u\left(0\right). \end{array}$$

When the control quantity *u* is limited by |*u*| ≤ *r*, the fact that all initial positions of the origin can be reached in *k* steps calls the *k* equal time zone *G*(*k*). The *u*(0) can be calculated.

(7) $$\begin{array}{c} u=\frac{x_{2}+y/h}{h},\;\left|x_{2}+y/h\right|\leq hr,\;\left|y\right|\leq h^{2}r. \end{array}$$

The optimal control should be *u* = *r*, so we can deduce the following equation:

(8) $$\begin{array}{c} u=-r\left(\frac{x_{2}+hy}{h}\right),\;\left|x_{2}+y/h\right|>hr,\;\left|y\right|\leq h^{2}r. \end{array}$$

According to formulas (7) and (8), we can get the following equation:

(9) $$\begin{array}{c} \left\{\begin{array}{c} u=-r\text{sat}\left(\left(x_{2}+\frac{y}{h}\right),hr\right),\;\left|y\right|\leq h^{2}r,\\ \text{sat}\left(x,\lambda \right)=\left\{\begin{array}{c} \text{sign}\left(x\right),\;\left|x\right|>\lambda ,\\ \frac{x}{\lambda },\;\left|x\right|\leq \lambda . \end{array}\right) \end{array}\right) \end{array}$$

Then, the expression of fast optimal control synthesis function can be obtained from expressions (5)–(9). So, fst definition is as follows:

(10) $$\begin{array}{c} \text{fst}\left(x_{1}\left(k\right)-r\left(k\right),x_{2}\left(k\right),r_{1},h_{1}\right)=\left\{\begin{array}{c} -r_{1}\cdot \text{sign}\left(a\right),\;\left|a\right|>d,\\ -r_{1}\cdot \left(\frac{a}{d}\right),\;\left|a\right|\leq d, \end{array}\right) \end{array}$$

(11) $$\begin{array}{c} a=\left\{\begin{array}{c} a_{0}=\sqrt{d^{2}+8r_{1}\left|m\right|},\\ x_{2}\left(k\right)+\frac{a_{0}-d}{2}\cdot \text{sign}\left(m\right),\;\left|m\right|>d_{0},\\ x_{2}\left(k\right)+\frac{m}{h_{1}},\;\left|m\right|\leq d_{0}, \end{array}\right) \end{array}$$

where *r*_1_ and *h*_1_ are TD parameters, *r* (*k*) is the input signal, *d* = *r*_1_*h*_1_, *d*_0_ = *dh*_1_, and *m* = *x*_1_(*k*) + *h*_1_*x*_1_(*k*).

Parameter *r*_1_ is the speed factor that can define the tracking speed. When *r*_1_ is faster, the tracking speed is greater. Parameter *h*_1_ is the filtering factor. But parameter *r*_1_ and parameter *h*_1_ are mutual restrictions, and they should manage to coordinate and cooperate with each other.

Formula (10) is a time-best solution which can ensure the fastest convergence from *x*_1_ to input signal without any overshoot. When formulas (10) and (11) are applied for the purpose of defining the transient profile, *r*_1_ and *h*_1_ can be changed individually by the desired control speed and smoothness. Based on tracking differentiator and arranging transition processes, the error signal of the transition process can be tracked.

ESO is hard to have an accurate model, so the nonlinear feedback is used to construct the observer. The ESO state space equation can be given as follows:

(12) $$\begin{array}{c} \left\{\begin{array}{c} {\dot{x}}_{1}=x_{2},\\ \vdots \\ {\dot{x}}_{n-1}=x_{n},\\ {\dot{x}}_{n}=f\left(x_{1},x_{2},\ldots ,x_{n}\right)+b_{1}u,\\ y=x_{1}. \end{array}\right) \end{array}$$

The typical differential equation of ESO can be given as follows:

(13) $$\begin{array}{c} \left\{\begin{array}{c} \;\varepsilon =\;z_{1}-y,\\ {\dot{z}}_{1}=z_{2}-\beta_{1}\varepsilon ,\\ \vdots \\ {\dot{z}}_{n}=z_{n+1}-\beta_{n}\text{fal}\left(\varepsilon ,a_{n-1},\delta_{n-1}\right)+b_{1}u\left(k\right),\\ {\dot{z}}_{n+1}=-\beta_{n+1}\text{fal}\left(\varepsilon ,a_{n},\delta_{n}\right), \end{array}\right) \end{array}$$

where *ε* is the estimation error; *n* is the control system order. Parameters *a*, *δ*, and Β are ESO parameters. *z* is the observations of the tracking state variables. fal is the nonlinear function.

The discrete ESO mathematical model is given as follows:

(14) $$\begin{array}{c} \left\{\begin{array}{c} z_{1}\left(k+1\right)=z_{1}\left(k\right)+H_{1}\left(z_{2}\left(k\right)-\beta_{1}\cdot \varepsilon \left(k\right)\right),\\ z_{2}\left(k+1\right)=z_{2}\left(k\right)+H_{2}\left(z_{3}\left(k\right)-\beta_{2}\cdot \text{fal}\left(\varepsilon \left(k\right),a_{1},\delta_{1}\right)\right),\\ z_{3}\left(k+1\right)=z_{3}\left(k\right)+H_{3}\left(z_{4}\left(k\right)-\beta_{3}\cdot \text{fal}\left(\varepsilon \left(k\right),a_{2},\delta_{2}\right)+b_{1}u\left(k\right)\right),\\ z_{4}\left(k+1\right)=z_{4}\left(k\right)-H_{4}\left(\beta_{4}\cdot \text{fal}\left(\varepsilon \left(k\right),a_{3},\delta_{3}\right)\right), \end{array}\right) \end{array}$$

where *z*_1_(*k*) to *z*_4_(*k*) are output signals of ESO, *k* is the sampling number, *ε*(k) = *z*_1_(*k*)-*y* (*k*), *y* (*k*) is the system output signal, *H*_1_ to *H*_4_ are ESO parameters, *β*_1_ to *β*_4_ are ESO parameters, *δ*_1_ to *δ*_3_ are ESO parameters, *b*_1_ is ESO parameter, and *b*_1_ = 0, *a*_1_ = 0.5, *a*_2_ = 0.25, and *a*_3_ = 0.125, *β*_1_ to *β*_4_ are ESO parameters, fal is a control function, and its definition is as follows:

(15) fal εk,a,ii=1,2,3δii=1,2,3=εkδi1−ai,εk>δi,signεkεkai,εk≤δi.

The NLSEF mathematical model in this paper is given as follows:

(16) $$\begin{array}{c} u_{0}\left(k\right)=\text{fst}\left(e_{1}\left(k\right),ce_{2}\left(k\right),r_{2},h_{2}\right)-\frac{z_{4}\left(k\right)}{b_{2}}, \end{array}$$

where *e*_1_(*k*) = *x*_1_(*k*) − *z*_1_(*k*), *e*_2_(*k*) = *x*_2_(*k*) − *z*_2_(*k*), *r*_2_, *h*_2_, *c*, and *b*_2_ are ESO parameters, *b*_2_ = 1.

**Figure 4:**
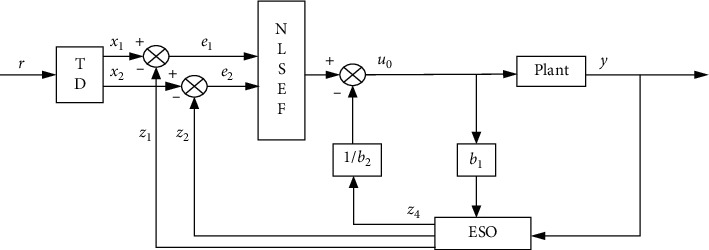
ADRC structure block diagram.

ESO is used for real-time estimation and disturbance compensation. In this way, the closed-loop system can be designed by the general error feedback method after the system is transformed into a linear integrator connected type. Therefore, the closed-loop system has satisfactory performances. Combining the nonlinear feedback combination and the total disturbance estimation, the third-order ADRC structure block diagram is shown in Figure 4, *y* is the output signal from the control system, *r* is the input signal, *x*_1_ and *x*_2_ are output signals of the TD, and *z*_1_ to *z*_4_ are output signals of the NLSEF. Two errors *e*_1_ and *e*_2_ are formed by the difference between signal *x*_1_, *x*_2,_ and signal *z*_1_, *z*_2_. Then, the control amount *u*_0_ is generated by the nonlinear function of *e*_1_, *e*_2_. Finally, the difference between the control amount *u*_0_ and *z*_2_ will drive the plant.

There are 16 parameters needed to tune in ADRC, including *r*_1_, *h*_1_, *r*_2_, *h*_2_, *H*_1_ to *H*_4,_*β*_1_ to *β*_4,_*δ*_1_ to *δ*_3_, and *c*. ADRC controller parameters greatly influence system response speed, dynamic controllability, and system performances. Therefore, it is crucial to get appropriate ADRC parameters to achieve a high maneuverability system. Parameter *h* is the precision parameter that can decide the system aggressiveness and it is usually a multiple of the sampling period by a factor of at least four [13]. In this paper, the sampling period is in the range of [0.01 0.25]. So, in this paper set *h* is equal to 1. Too large or too small *b*_1_ can increase disturbances for ESO. To weaken the overshoot, the mechanism wear, and the unnecessary energy loss caused by a given large change, it is important to arrange the appropriate procedure according to the bearing capacity of the object [13]. When the changing of the controlled object is not very drastic, the fact that parameter *b*_1_ is equal to 0 can simplify the controller structure.

There are a lot of articles for convergence and stability analyses of ADRC controllers [56]. In the literature [57], the ADRC convergence was proved for a class of the single-input-output nonlinear system. The literature [58] proved the ADRC convergence in the multiple input-output systems. In the literature [59], the ADRC convergence was proved for the lower triangular uncertain system. The ADRC stability analyses mainly include the limit cycle analysis, the absolute stability analysis, and the Lyapunov stability analysis based on the description function method [56]. In the literatures [60–62], the limit cycle analysis of the ADRC with single and double nonlinear links in ESO was studied by using the description function method. In the literatures [63–65], the absolute stability of the nominal system and the robust absolute stability were considered.

## 4. The Proposed Control Method

### 4.1. Lévy-Flight Beetle Antennae Search Algorithm

BAS is a new metaheuristic algorithm that mimics the beetle foraging behavior. One beetle possesses two long antennae that are longer than its body, and the antennae are used to detect food and potential mates. If one antenna is closer to the food resources, the beetle moves to that side, which corresponds to a stronger food odor. Through a series of circular movements, the beetle finds the food resources. In BAS, the moving behavior is expressed as a mathematical model to solve optimization problems. In the basic BAS, the search lengths and steps are fixed, which facilitates the global search in the early phase. However, the use of fixed search lengths and steps in the latter phase deteriorates the global search process, and less information regarding the optimal solution is available. The Lévy-flight beetle antennae search algorithm (LBAS), which uses the Lévy-flight mechanism and self-learning strategy, can be applied to the basic BAS to enhance its search diversity and performance. In 1925, the French mathematician Lévy proposed the Lévy-flight mechanism. The Lévy-flight method is a finite-velocity random walk involving steps designed based on a fixed time and dynamical movement procedure. Because the Lévy-flight method is a scale-invariant mathematical model whose long steps can be relocated by small steps, the LBAS can not only minimize the number of search iterations but also prevent the algorithm from falling into local solutions. When the Lévy-flight method is introduced in the beetle antennae search algorithm, the enhanced algorithm can realize a satisfactory search exploration and demonstrate excellent convergence accuracy and optimized predation position. The self-learning strategy, which works by regulating the displacement difference between the optimal solution and the individual solution in each iteration, can weaken the high randomness and the stochastic blindness of the heavy-tailed distribution in the Lévy-flight mechanism. With time continuing, the position adjustment can get more and more subtle, and the searching scope is gradually changed from large to small. To enlarge the initial searching exploration, the initial beetle moving orientation is set to shift randomly. The LBAS process can be described as follows:

Step 1. Initially, set the *D*-dimensional finding scope, set the maximum number of search iterations *T*, generate *N* beetles positions *x*_*i*_^*t*^, *i* is *i*-th beetle position, define the searching scope, and set the current iteration *t* which equals 1.
Step 2. The head orientation of one beetle is random at the beginning of iteration because the beetles are in an unknown environment. A vector with a random direction can be set to normalize in any searching dimension.
  (17) $$\begin{array}{c} \overset{⟶}{b}=\frac{\text{rnd}\left(D,1\right)}{\left\|\text{rnd}\left(D,1\right)\right\|}, \end{array}$$
where rnd(·) means a random function.
Step 3. Beetles apply two antennae to find food resources. The beetle will move to the left side if the left antenna side receives a stronger food odor. The beetle will go to the right side according to the same strategy.
  (18) $$\begin{array}{c} x_{ri}^{t}=x_{i}^{t}+d^{t}\overset{⟶}{b}, \end{array}$$
  (19) $$\begin{array}{c} x_{li}^{t}=x_{i}^{t}-d^{t}\overset{⟶}{b}, \end{array}$$
where *x*_*ri*_^*t*^ and *x*_*li*_^*t*^ mean the right antenna position and the left antenna position in *i*-th beetle, respectively. *d*^*t*^ is the antennae sensing length.
Step 4. The beetle will update its next position according to the detected odor. Therefore, we can find the next position depending on difference between the right and left positions. The next beetle position can be updated by
  (20) $$\begin{array}{c} x_{i}^{t+1}=x_{i}^{t}+\overset{⟶}{b}\delta^{t}\text{sign }\left(f\left(x_{ri}^{t}\right)-f\left(x_{li}^{t}\right)\right), \end{array}$$
  (21) $$\begin{array}{c} \delta^{t}=s\left|x_{i}^{t}-x^{*}\right|, \end{array}$$
where *x*_*ri*_^*t*^ and *x*_*li*_^*t*^ are the right antenna position and the left antenna position in *i*-th beetle, *x*^*∗*^ means the food, *d*^*k*^ is the antennae sensing length, sign(·) represents a sign function, and *s* is the Lévy random step.
  (22) $$\begin{array}{c} s=\frac{\mu }{{\left|v\right|}^{\left(1/\beta \right)}}, \end{array}$$
where *β* is the power-law exponent. *μ* ~ *N*(0, *σ*_*μ*_^2^), *v* ~ *N*(0, *σ*_*v*_^2^), *σ*_*v*_=1, *σ*_*μ*_ can be described as
  (23) $$\begin{array}{c} \sigma_{\mu }={\left(\frac{\Gamma \left(1+\beta \right)\cdot \sin\left(\pi \beta |/|2\right)}{\Gamma \left(\left(1+\beta \right)|/|2\right)\cdot \beta \cdot 2^{\left(\left(\beta -1\right)|/|2\right)}}\right)}^{1/\beta }. \end{array}$$
Step 5. The sensing length should have not only the strong searching ability in the early phase, but also meticulous convergence precision in the later phase, so the sensing length should be added to the random disturbance which can make the searching scope from large to small. The next sensing length can be updated by
  (24) $$\begin{array}{c} d^{t+1}=w\cdot d^{t}+0.01, \end{array}$$
where *w* is the reduction factor in the range of [0, 1].
Step 6. Calculate all fitness values. Select and replace the optimal solution and the best fitness value if there is a better solution, compared with other fitness values. Replace the optimal solution and the best fitness value if there is a better fitness value. Record the global optimum solution and fitness value.
Step 7. Judge whether the optimization circumstances meet the end condition. If not, return to Step 2 and go on. Otherwise, stop iterative loops.

The LBAS can be summarized in the pseudocode shown in Algorithm 1.

### 4.2. System Control Strategy

Evaluation functions mainly include the integral of the squared value of error (ISE, integration-square value-error), the mean of the square of the error (MSE, mean-square value-error), the integration of the absolute value of error (IAE, integration-absolute value-error), and the integral of time multiplied by the absolute value of error (ITAE, integration-time-absolute value-error). IAE and ISE both are the single objective functions; the single factor function cannot reflect complex hydraulic systems states. MSE computes the average of the ISE and time, which can minimize the shortcomings of the ISE. But the system has to drive for a long time to calculate the average of the ISE and time, punish large errors, and accumulate small errors that will damage the control system in the later stages. ITAE can weaken long-term errors and applies the additional time-multiplication to reach a good system threshold and utilization, which endures long-time errors and makes excellent dynamic performances.

(25) $$\begin{array}{c} \text{ITAE}=\int_{0}^{\infty }t\left|e\left(t\right)\right|\text{d}t. \end{array}$$

The ADRC control performances depend on sixteen parameters. To get the best ADRC controller, the ADRC tuning problem is converted into a class of sixteen-dimensional searching questions in this paper. The ADRC working principle in this paper is that ADRC controls the system by applying sixteen parameters that are tuned by LBAS, and the ITAE value will be automatically computed using LBAS. ADRC parameters can be represented as a feasible vector solution that is coded by a real number. ADRC sixteen parameters can be seen as each position in sixteen-dimensional space. The ITAE can be seen as the evaluation function. First, beetle positions can be randomly set in a sixteen-dimensional space. Then, beetle positions are input into the ADRC controller as sixteen parameters. Each position that minimizes the ITAE can be seen as the optimum ADRC parameters, and it is used to update the optimum ADRC parameters. If the control system performance can meet all requirements in the engineering fields or the searching procedure can reach the maximum iteration number, the best beetle position will be selected as the final ADRC parameters. ADRC parameters tuning steps are shown as follows:

  Step 1. Initially generate *N* beetles positions *x*_*i*_^*t*^, set the sixteen searching dimensions, the maximum number of searching iterations *T*, and all initial algorithm parameters, and set *t* = 1, the searching scope. Each beetle position can be converted into sixteen ADRC parameters to calculate ITAE.
  Step 2. Define the searching direction in sixteen-dimensional space using (17) to expand the initial exploration environment. In nature, one beetle does not know the food position when foraging and applies its antennae to set the next position. When each antenna on one side is closer to the searching aim, the searching aim odor received by the antenna is stronger, and the beetle will move to that side. Get the beetle right-left position in (18) and (19). If the antenna side is closer to the smaller ITAE, the beetle will go to the antenna side.
  Step 3. Operate the control system. Right and left antennae positions of the beetle can be seen as sixteen ADRC parameters and are put into the ADRC controller. Calculate the ITAE values of the right antenna position and the left antenna position. Calculate the ITAE values of the right antenna position and the left antenna position. Update the next beetle position in (20) to get a new set of beetle positions. Then, update the next sensing length in (24). By updating, the next sensing length of the antennae will be carried over to the next generation.
  Step 4. Operate the control system. All updated beetle positions will be new ADRC parameters which will be taken into the fitness function to calculate the ITAE value. Calculate the ITAE of each beetle in the control system. Compare all ITAE values and find the current minimum ITAE. After comparing the current minimum ITAE with the previous minimum ITAE, the global optimum best position can be updated, and the global optimum beetle position is selected to be the best ADRC parameters. The beetle position which minimizes the ITAE can be seen as the current optimum solution. Update the global minimum ITAE and global optimum position.
  Step 5. Calculate *t* = *t* + 1. Judge whether iterations meet terminating conditions *t* = *T*. If *t* meets the terminating condition, the global optimum beetle position can be seen as the final ADRC parameters. If not, return to Step 2 and go on iterations.

**Figure 5:**
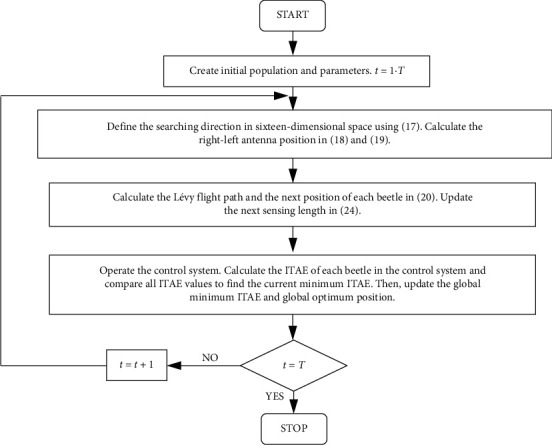
Working flow chart of the LBAS-ADRC controller.

The flow chart for the LBAS-ADRC controller working steps is shown in Figure 5.

The fact that ADRC parameters are difficult to be adjusted affects the control abilities of hydraulic systems; an optimization algorithm LBAS is used to adjust ADRC parameters of hydraulic systems in this paper. To solve the problem that BAS will fall into local solutions and stop in the later iterative process, the Lévy-flight strategy is introduced to enhance the basic BAS searching ability. A third-order ADRC controller having sixteen parameters is designed based on LBAS. And ADRC parameters tuning problem was converted into the sixteen-dimensional problem. This paper takes hydraulic systems of the quadruped robot as the controlled object to verify that ADRC optimized by LBAS has higher control accuracy and antidisturbance ability than other compared algorithms and effectively improves dynamic performances of the controlled system.

## 5. Benchmark Function Discussion

### 5.1. Parameters and Environments

Benchmark functions can indicate the searching performance of the algorithm. And this paper uses different functions to test the searching performance of the proposed algorithm. Literature [45] tested ten functions, so this paper selected different functions including six low-dimensional functions (*f*_1_–*f*_6_) and two high-dimensional functions (*f*_7_-*f*_8_) in Table 1. In Table 1, *D* means the searching dimension. Scope means the searching range. Aim means the ideal value. Different algorithms include LBAS, particle swarm optimizer (PSO) [66], Genetic Algorithm (GA) [67], and Flower Pollination Algorithm (FPA) [68]. For PSO, *c*_1_ = *c*_1_ = 1 and *w*=1. For GA, the selection probability was equal to 0.8, the crossover probability was equal to 0.8, and the mutation probability was equal to 0.2. For FPA, the switching probability was equal to 0.8, and the power-law exponent was equal to 1.5. For the LBAS, the power-law exponent *β* = 1.5. All algorithm details can be found in the original algorithm literature. The maximum number of iterations was set to 500, and the population size was set to 20. All algorithms were tested for ten independent runs. To make a fair comparison, all algorithms were programmed in MATLAB. All experiments were conducted on a laptop with Intel Core i5-4210U CPU, 2.30 GHz, 4 GB RAM. All data and figures were completed in MATLAB.

### 5.2. Testing Results Discussion

To show the searching performance of LBAS, this paper gives four indicators including the smallest calculation result (Min), the worst calculation result (Max), the median (Med), and the standard deviation (Std) in Table 2. Note that all calculation results discussed in this paper are ten independent runs. Median means the value in the middle of the sequence when all values in the statistical population are arranged in order of size to form a sequence. Med is not affected by large or small data. In many cases, it is more appropriate to use for representing the general level of all data. The standard deviation is the arithmetic square root of the arithmetic mean of the mean-square deviation, and it can reflect the discrete degree of all data. Large Std has a large difference between most values and the average value. LBAS has the testing value of all results, which show that the proposed algorithm has better-searching precision than other compared algorithms. In other words, the fact that LBAS has a small Std shows that the calculated value by LBAS is closer to their average values. All searching results display that LBAS owns good stability for finding the aim value.

### 5.3. Iteration Results in Discussion

**Figure 6:**
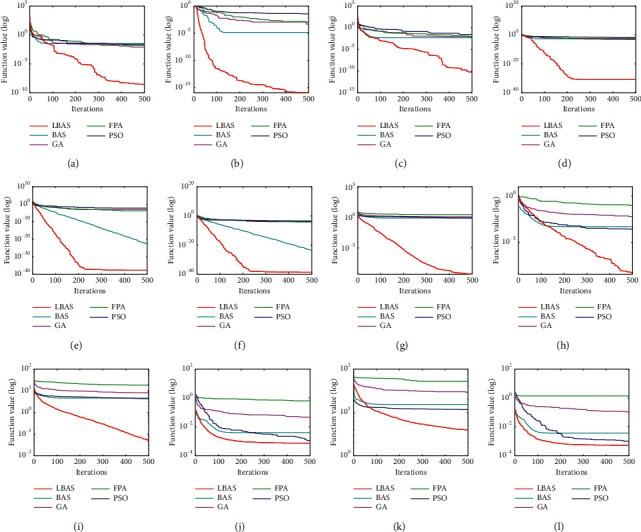
Convergence curves for functions. (a) *f*_1_. (b) *f*_2_. (c) *f*_3_. (d) *f*_4_. (e) *f*_5_. (f) *f*_6_. (g) *f*_7(*D*=20)_. (h) *f*_8(*D*=20)_. (i) *f*_7(D=50)_. (j) *f*_8(*D*=50)_. (k) *f*_7(*D*=100)_. (l) *f*_8(D=100)_.

Iteration is a feedback process to find the desired goal. Each repetition of all processes is called one iteration, and its result can be seen as the initial value for the next iteration. To show the convergence ability of the proposed algorithm, the average convergence log curves of all algorithms used for different functions are shown in Figure 6. Note that all convergence curves discussed in this paper are the averages of ten independent runs. Searching performances and the iteration speed are better than those of other algorithms. As the dimension increases, the searching performance degradation of compared algorithms is violent. LBAS can give the largest iteration speed and highest efficiency when finding the function aim. In other words, LBAS not only gives fewer iterations to find the best aim but also owns better performance than compared algorithms. Iterations show that the proposed algorithm can strengthen the iteration speed and global-local searching ability of basis BAS.

### 5.4. Boxplot Results Discussion

**Figure 7:**
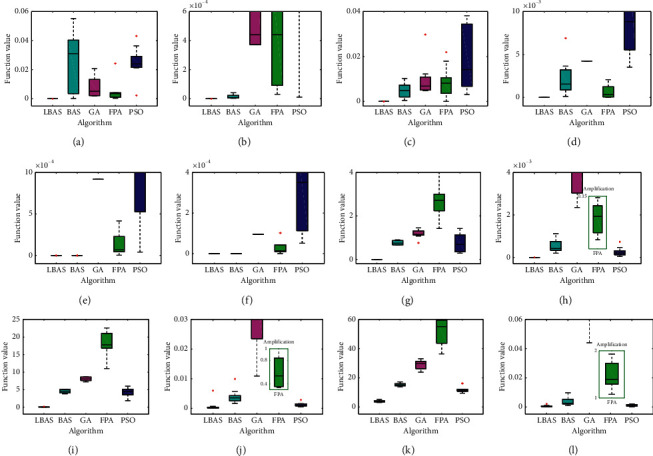
Boxplot charts of functions. (a) *f*_1_. (b) *f*_2_. (c) *f*_3_. (d) *f*_4_. (e) *f*_5_. (f) *f*_6_. (g) *f*_7(*D*=20)_. (h) *f*_8(*D*=20)_. (i) *f*_7(D=50)_. (j) *f*_8(*D*=50)_. (k) *f*_7(*D*=100)_. (l) *f*_8(D=100)_.

Boxplot can provide some key information about the data location and dispersion, especially when different amounts of data are compared, and it can give six data nodes by arranging a group of data from large to small and calculating upper edge, the upper quartile, median, lower quartile, lower edge, and outlier. Boxplot can analyze data symmetry and distribution performances.  Figure 7 shows boxplot charts of all algorithms when calculating different functions after 10 independent runs. Note that all boxplots discussed in this paper are the averages of ten independent runs. LBAS can give the narrowest boxplot charts and smallest outliers in all functions, and the median is lower than those of other values computed by other algorithms. It is clearly shown that LBAS can offer good performance for most functions as the dimension increases. For two-dimensional functions, PSO has the worst boxplot and the maximum difference between the upper quartile and the lower quartile. For high-dimensional functions, FPA has the worst boxplot. Boxplot can show that the proposed algorithm can give high stability and good searching performance.

## 6. Results and Discussion

### 6.1. System Parameters and the Application Object

**Figure 8:**
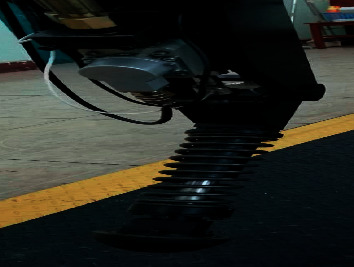
Single leg of the hydraulic quadruped robot.

All controlled systems include one-upper computer and four-lower computers, and one-upper computer controls three active joints of the single leg. The upper computer hardware mainly includes the CAN-BUS module and the analog acquisition module. The lower computer mainly has the analog-to-digital converter, the digital to analog conversion, and the CAN-BUS module. The robot applies the fixed pump station for the oil supplying and the pump and accumulator are combined to supply oil to reduce the heating of the system. The fuel supplying pressure sensors monitor the pressure-flow changing for the robot airborne. The proposed ADRC controller was used for the knee-joint position control system of the hydraulic quadruped robot developed by the Hydraulic Quadruped Robot Lab at Harbin University of Science and Technology. The displacement sensor is the LVDT-PA1HL60X sensor designed in the Fuxin-Li Sheng Automatic Control Co., Ltd., Fuxin, China. The servovalve, which is designed by the 18th Research Institute of the First Academy of China Aerospace Science and Technology Corporation, Beijing, China, is the SFL212F-12/8-21-40 force-feedback valve. The controlled system was shown in Figure 8.

To show the control ability of the proposed ADRC controller, this paper tested different ADRC controller parameters by simulating different algorithms in MATLAB. Initial parameters of all algorithms were chosen according to all basic algorithm literatures, and all details of algorithms can be seen in basic literature. ITAE was chosen as the evaluation function. Set maximum iterations 200. Set population size 20. Set *H*_1_ to *H*_4_ in the range of [0, 0.5]. Set other ADRC parameters in searching range of [1*E* − 50 100000]. Each algorithm was implemented in MATLAB software.

### 6.2. Iteration Curves and Boxplots Analysis

**Figure 9:**
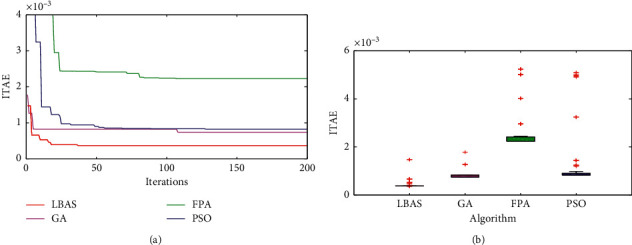
Iteration curves and boxplot. (a) Iteration curves; (b) boxplots.

Figure 9 shows algorithm iteration curves and boxplots. The iteration aims to search the ideal value by repeating feedback actions; the current found value can be seen as the starting value of the next iteration.  Figure 9(a) shows four iteration curves of different algorithms computing the evaluation function. In Figure 7(a), LBAS has the biggest iteration speed in all algorithms, which exhibits that LBAS can improve the feasible solution diversities compared to the other three algorithms. GA curve has the strongest iteration ability in the early iteration phase, but GA is in the precocity state and will fall into the local feasible solution in the later iteration. The PSO iteration speed is slow in the early stage. FPA has the worst iteration performance and the lowest searching accuracy. The boxplot chart, which can exhibit a set of dispersed data, is technical graphics and can describe key standards. The data relevance and some special values support data analysis. There are five parts in a boxplot chart, including the minimum, the maximum, the median, and the upper-lower quartiles. The system robustness can be shown by different boxplot charts. In Figure 9(b), LBAS has the fewest outliers and the narrowest form, which shows that the ADRC tuned by LBAS owns the balance ability. FPA has the maximum outlier and the most dispersed data. The boxplot chart indicates that LBAS-ADRC has excellent control performance.

### 6.3. Time Domain Characteristic Analysis

Time domain analysis, which is accurate and easy to accept, is to analyze the system performance according to the step response curve and the temporal response of the controlled system. Under the actions of input signals, changed procedures of system output signals are called the temporal response. The temporal response usually consists of two parts including the system transient response and the system steady-state response. The transient response refers to the changed signal process from an initial state to a stable state under the actions of some input signals. The steady-state response refers to the system's final state when the time approaches infinity. The transient response mainly has four indices including overshoot *M*_*p*_, peak time *t*_*p*_, settling time *t*_*s*_, and delay time *t*_*d*_; overshoot *M*_*p*_ is the ratio of the instantaneous maximum deviation to the final steady-state value, peak time *t*_*p*_ indicates the required time that makes the system output reach the maximum value, and delay time *t*_*d*_ means the required time that makes the system output reach the half-steady state. Five percent to two percent of the steady-state value is taken as the error range, and the running time that reaches and remains within the error range is called the adjustment time. The system steady-state response *e*_r_ is defined as the difference between the expected output signal and the actual output signal. And this paper gives the final tuned parameters value for ADRC followed by sixteen ADRC parameters.  Table 3 displays ITAE values and indices of temporal response.

For LBAS: [*r*_1_*h*_1_*r*_2_*h*_2_*c δ*_1_*δ*_2_*δ*_3_*β*_1_*β*_2_*β*_3_*β*_4_*H*_1_*H*_2_*H*_3_*H*_4_] = [0.0038 5520.6063 100000 21130.5851 84775.9693 3953.5879 100000 5499.5581 100000 3376.3183 16021.5065, 14175.2430 0.5 0.5 0.0098 0.4334].

For GA: [*r*_1_*h*_1_*r*_2_*h*_2_*c δ*_1_*δ*_2_*δ*_3_*β*_1_*β*_2_*β*_3_*β*_4_*H*_1_*H*_2_*H*_3_*H*_4_] = [0.0441 2541.0295 8135.2502 3273.1755 7094.0562 3292.8594 2448.7805 5473.1135 9973.8502 4261.6313 7585.8044 1248.4086 0.0946 0.0465 0.0974 0.0242].

For FPA: [*r*_1_*h*_1_*r*_2_*h*_2_*c δ*_1_*δ*_2_*δ*_3_*β*_1_*β*_2_*β*_3_*β*_4_*H*_1_*H*_2_*H*_3_*H*_4_] = [2.2488*E* − 07 99999.9709 99999.9550 99999.9562 99999.9550 0.0439 99999.9551 99999.9555 0.0452 0.0585 99999.9511 0.04517 2.2577*E* − 07 2.2281*E* − 07 4.8196*E* − 07 2.2066*E* − 07].

For PSO: [*r*_1_*h*_1_*r*_2_*h*_2_*c δ*_1_*δ*_2_*δ*_3_*β*_1_*β*_2_*β*_3_*β*_4_*H*_1_*H*_2_*H*_3_*H*_4_] = [0.4827 976.4213 124.8167 562.0781 996.4221 148.7752 669.7964 737.8845 117.6908 1*E* − 50 33.9119 111.4523 1*E* − 50 0.5 1*E* − 50 0.5].

In Table 3, LBAS-ADRC has the smallest ITAE than other control methods, which exhibits that the controlled system has good performance and competitive advantage. For the system transient response, as can be shown in Table 3, the ADRC controller tuned by the LBAS owns the smallest peak time, settling time, and delay time. Although the overshoot of GA-ADRC is lower than the overshoot of LBAS-ADRC, GA-ADRC, which needs to run a long time to achieve the steady state, has the biggest peak time, the maximum settling time, and the highest delay time, which can destroy controlled stability resulting in bad consequence. For the system steady-state response, the steady-state error *e*_*r*_ reflects system abilities of eliminating the dead-zone character. Due to the existence of dead-zone in systems, when the modulation frequency is very high and the speed is low, output signals of systems have large oscillatory harmonics, which can lead to a strong pulse wave and even cause system disorientation. LBAS-ADRC has the minimum steady-state error, which shows that the ADRC control strategy combining LBAS has an effective reduction in surplus in the system, and can better control the steady-state error.

**Figure 10:**
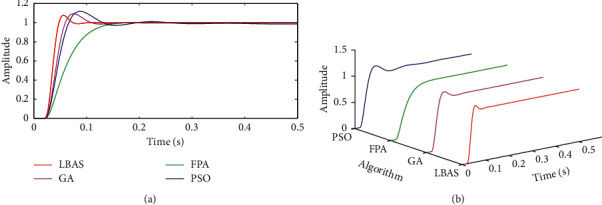
Step response curves. (a) Two-dimensional curves; (b) three-dimensional curves.

Figure 10 shows all step response curves. ADRC derived by LBAS has the fastest response speed and fastest reach of the ideal value in all control algorithms. The LBAS-ADRC controller owns some overshoot, but the ADRC controller will regulate the output signal to the ideal signal value. The FPA-ADRC controller takes a long time to reach a steady state. As time goes by, the working conditions of most environment are changed; FPA-ADRC controller can cause the performance degradation of controlled systems, which can cause system damage. PSO-ADRC controller has the maximum overshoot. A maximum overshoot system has bad transient performance and will cause oscillation sharp occurring, not meeting the engineering requirements. The step response curve demonstrates that LBAS-ADRC is effective in maintaining the system precision, limiting the system overshoot, and weakening the system transient phenomenon.

The ESO can observe generalized disturbance constituent parts in real time, including model discrepancies and exogenous disturbances. And ESO can compensate for unpredicted disturbances in the control signal. ESO was autonomous in the mathematical model and introduced within the ADRC framework. So, ESO can be seen as an important part of modern controls. The advantage of ESO is that it is not necessary to know whether the disturbance function is continuous or changed in the actual control system. The ESO core is the disturbance estimation and the compensation, which observes the external-internal disturbances. Therefore, the controlled system can be simplified into a series system by the disturbance compensation when the controlled system is linear or nonlinear, time-varying or time invariant.

### 6.4. Frequency Domain Characteristic Analysis

When the frequency of the input sinusoidal signal is changed continuously from zero to infinity, the amplitude ratio *A* of the steady-state amplitude to the ideal amplitude is called the amplitude-frequency characteristic, and the phase difference *φ*_*ω*_ between the steady-state output signal and the input signal is called the phase-frequency characteristic. If *A* is closer to 1 and *φ*_*ω*_ is closer to 0, the system has good performance. The frequency characteristics reflect the stability and dynamic and anti-interference abilities of controlled systems. For input sinusoidal signals, the angular velocity, respectively, selected 10, 15, 20, 25, 30, and 35, and the initial phase was zero, and the amplitude, respectively, selected 1, 5, 10, 15, 20, and 25.  Table 4 shows the frequency characteristic parameter. To more clearly display the amplitude-frequency characteristic, this paper computed the absolute value of the difference between 1 and the amplitude-frequency characteristic index.  Table 4 shows ∆*A*_*ω*_ = |1 − *A*_*ω*_|. From Table 4, it can be seen that the amplitude-frequency characteristic of the ADRC tuned by the LBAS is the closest to 1 and that the phase-frequency characteristic of the LBAS-ADRC is the closest to 0.

**Figure 11:**
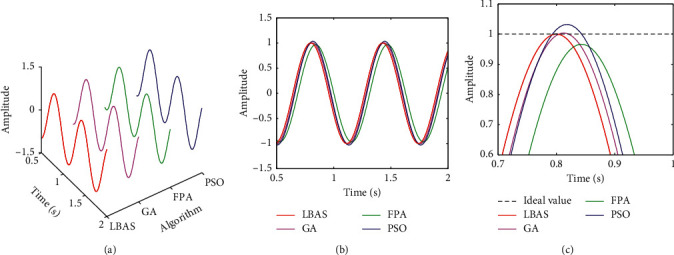
Sinusoidal curves of amplitude 1 and angular velocity 10. (a) Three-dimensional curve; (b) two-dimensional curve; (c) local amplification curves.

**Figure 12:**
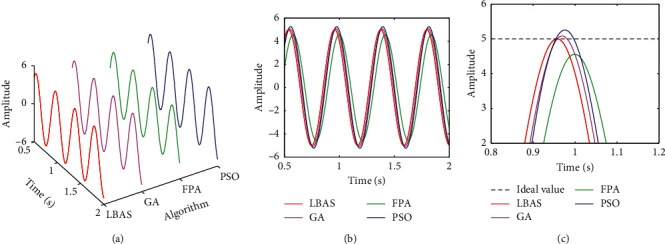
Sinusoidal curves of amplitude 5 and angular velocity 15. (a) Three-dimensional curves; (b) two-dimensional curves; (c) local amplification curves.

**Figure 13:**
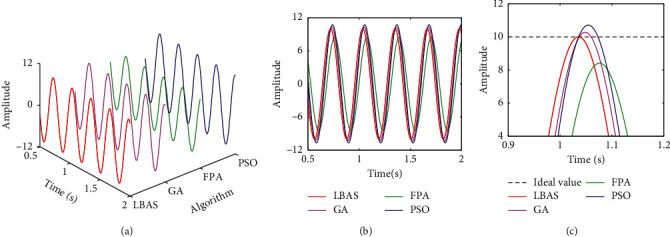
Sinusoidal curves of amplitude 10 and angular velocity 20. (a) Three-dimensional curves; (b) two-dimensional curves; (c) local amplification curves.

**Figure 14:**
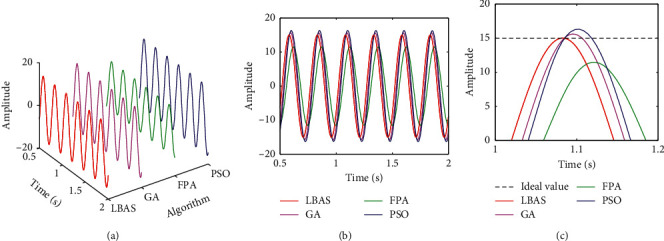
Sinusoidal curves of amplitude 15 and angular velocity 25. (a) Three-dimensional curves; (b) two-dimensional curves; (c) local amplification curves.

**Figure 15:**
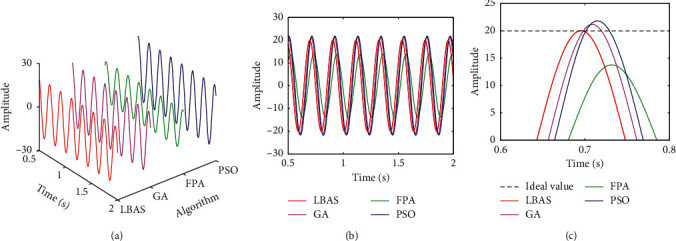
Sinusoidal curves of amplitude 20 and angular velocity 30. (a) Three-dimensional curves; (b) two-dimensional curves; (c) local amplification curves.

**Figure 16:**
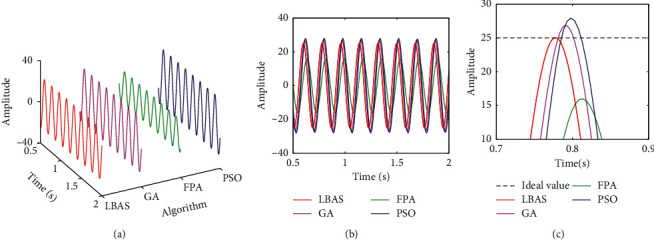
Sinusoidal curves of amplitude 25 and angular velocity 35. (a) Three-dimensional curves; (b) two-dimensional curves; (c) local amplification curves.

Figures 11–16, respectively, display all sinusoidal curves and the local amplification curves. The amplitudes of GA and PSO are larger than the ideal value, and the amplitudes of FPA are far lower than the ideal value. The LBAS-ADRC controller is closest to the ideal value. With the increase of angular velocity, except for the LBAS-ADRC controller, amplitude differences and system oscillations of other algorithms are larger and larger, and the amplitude difference of the FPA-ADRC controller is largest. LBAS-ADRC controller has satisfactory sinusoidal waveform, high dynamic response, great load characteristic, and wonderful precision. Local amplifications display that the LBAS-ADRC controller has vibration damping, suppression, and strong stiffness. Under the abnormal interference environment, the LBAS-ADRC controller owns the wonderful ability of keeping the reliability and preventing signal interference.

### 6.5. Ramp Signal Characteristic Analysis

**Figure 17:**
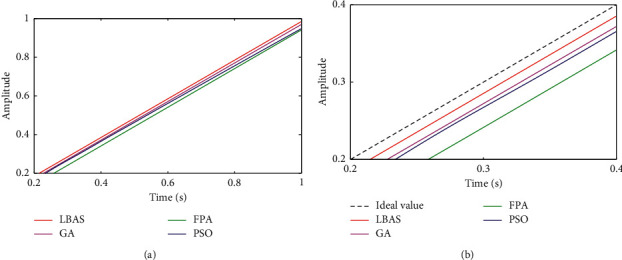
Ramp response curves of slope 1. (a) Two-dimensional curves; (b) local amplification curves.

**Figure 18:**
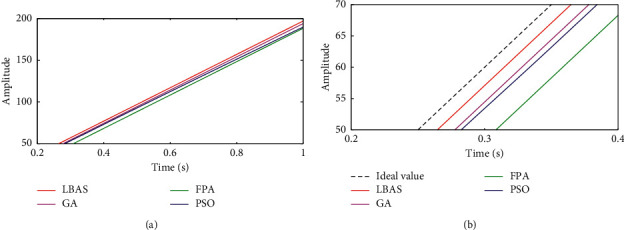
Ramp response curves of slope 200. (a) Two-dimensional curves; (b) local amplification curves.

The ramp signal is applied to analyze the system model and other pieces of information. In the negative half axis, the ramp signal is equal to zero. In the positive half axis, the ramp signal is a positive proportion function. The unit ramp signal is that the slope is equal to one. This paper used different ramp signals whose slopes were separately selected to be 1 and 200 to test the track and the orientation precision. The ramp signal testing results were displayed in Figures 17 and 18. From Figures 17 and 18, the controlled system has the lowest response speed and the largest error for FPA-ADRC. The LBAS-ADRC difference between the actual signal and the aim value is smallest in all figures when the signal slope increases gradually. Ramp response results show that LBAS-ADRC owns good dynamic response and show excellent balance, distinguished stability, and remarkable practicability.

Table 5 shows the ramp response characteristic. To show different ADRC control performances, this paper calculated slope values *S* and difference values ∆*S* between ideal values and actual values in Table 5. From Table 5, we can see that slope value *S* of the ADRC tuned by the LBAS is the closest to the ideal value and that the difference values ∆*S* of the LBAS-ADRC are the closest to 0. Table 5 shows that the proposed controller can keep the balance ability.

### 6.6. Linear Active Disturbance Rejection Control Analysis

**Figure 19:**
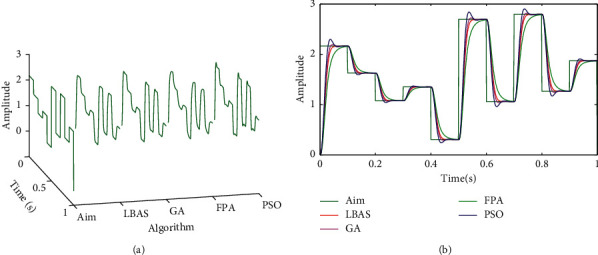
Random response curves 1 of LADRC. (a) Three-dimensional curves; (b) two-dimensional curves.

**Figure 20:**
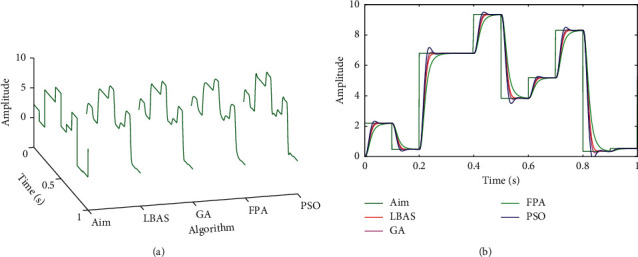
Random response curves 2 of LADRC. (a) Three-dimensional curves; (b) two-dimensional curves.

Based on the linear extended state observer, Zhiqiang Gao proposed the linear active disturbance rejection control (LADRC) [69]. In the LADRC, the TD part is omitted, and the controller focused on the ESO linear simplification and the nonlinear combined control equation. The ESO is linearized and its parameters are connected with the observer bandwidth to simplify ESO, which is called LESO. A simple PD control combination is given, and the proportional coefficient and differential-time constant are connected with the controller bandwidth to simplify the controller [70–72]. To illustrate the LBAS effectiveness in LADRC, this paper tested LADRC tuned by simulating different algorithms in MATLAB. In this paper, tuned parameters include the LADRC parameter *lb*_0_, the observer bandwidth *w*_0_, and two controller gains *k*_*p*_, *k*_*d*_. Set LADRC parameters in searching range of [1*E* − 50 100000]. To test performances of the LADRC, different response curves were got in Figures 19 and 20 when driving signals were random signals. Curves include three-dimensional curves and two-dimensional curves. From Figures 19 and 20, we can see that LBAS-LADRC has great anti-interference capabilities. Response curves of LBAS-LADRC are the closest to driving signals and own minimum overshoots. The curve of PSO-LADRC has the biggest oscillation. The curve of FPA-LADRC keeps away from the driving signal and has the signal distortion. The curve of GA-LADRC has a small amount of the signal overshoot. LADRC results show that LBAS has the great tuning ability in the LADRC.

### 6.7. Real-Time Environment Analysis

**Figure 21:**
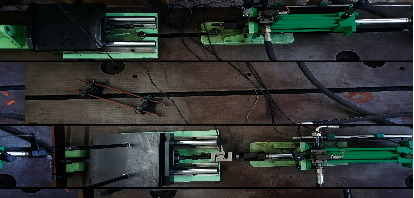
The experimental setup.

To show the performance of the LBAS-ADRC controller in the real-time environment, the proposed controller was applied in the real position control system of the semiphysical experiment platform. The hydraulic power was 5.5 k, the rated pressure was 5 MP, and the rated flow rate was 30 L/min. And *ADVANTECH PCL1710HG* was selected as the multifunction board; the base address is set to 300. System input signals will be got by the computer and transmitted to the system input port through the D/A conversion module of ADVANTECH PCL1710HG. At the same time, the displacement sensor feeds back the displacement signal and data; then, fed signals will be transmitted to another port from the A/D conversion module of *ADVANTECH PCL1710HG* two different channels.  Figure 21 is the experimental setup.

**Figure 22:**
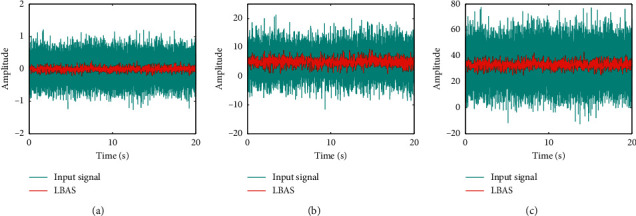
Oscillatory-disturbance response curves. (a) Signal 1; (b) signal 2; (c) signal 3.

To show the disturbance rejection capability of the proposed controller, this paper tested different oscillatory-disturbance signals based on the Gaussian distribution which is a random signal whose probability density distribution is normal. Oscillatory-disturbance signals can test the system performances of restraining irregular vibration. The testing results were displayed in Figure 22. Figure 22 displays that the LBAS-ADRC can enhance anti-interference ability whenever the system exhibits an oscillation or overshoot under tough circumstances. And, in an unknown environment, the proposed controller can give prominent stability and brilliant equilibrium. As the amplitude of the input signal increases, the proposed controller not only can reach the expected small-signal quickly but also can reduce the shaking and concussion. From the real-time environment analysis, we can see that the system exchanges information with the environment in the system operation. State variables information will be transmitted to outside parts, and some information is inhaled from outside parts; the system changes and develops in the information changing process with outside parts.

## 7. Conclusions

As a significant kind of power output equipment, hydraulic quadruped robots are commonly applied in automation and industry fields. In this paper, to improve the control performance and stability of the hydraulic quadruped robot, the ADRC controller tuned by the Lévy-flight beetle antennae search algorithm was used in the hydraulic servosystem of the robot. The proposed ADRC controller can keep the robustness, security, and invariability under the indeterminacy dynamic environment, which can greatly meet the requirement of the hydraulic control system. A hydraulic system model was given through theoretical analysis and scientific study. Meanwhile, GA, PSO, and FPA were applied to tune the ADRC parameter to compare the control abilities of the ADRC controller designed by different algorithms. Finally, all ADRC controller models were utilized for comparative results of the step response, the sinusoidal response, and the ramp signal. All analyses can be concluded that the proposed ADRC controller performance in both responses is undoubtedly greater than other ADRC controllers tuned by different algorithms regarding output chattering, tracking, and damping. In the future study, we will design a hybrid controller based ADRC controller and other control methods to improve the control stability of the hydraulic quadruped robot.

## Figures and Tables

**Algorithm 1:**
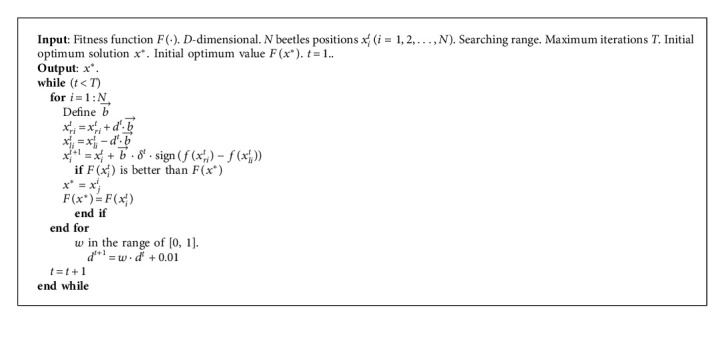
LBAS.

**Table 1:** Basic information on benchmark functions.

Name	Formulation	*D*	Scope	*A*_*im*_
Cube	*f*_1_(*x*)=100(*x*_2_ − *x*_1_^3^)^2^+(1 − *x*_1_)^2^	2	[−10, 10]	0
Himmelblau	*f*_2_(*x*)=(*x*_1_^2^+*x*_2_ − 11)^2^+(*x*_1_+*x*_2_^2^ − 7)^2^	2	[−10, 10]	0
Leon	*f*_3_(*x*)=100(*x*_2_ − *x*_1_^2^)^2^+(1 − *x*_1_)^2^	2	[−10, 10]	0
Lévy13	*f*_4_(*x*)= sin^2^(3*πx*_1_)+(*x*_1_ − 1)^2^[1+ sin^2^(3*πx*_2_)]+(*x*_2_ − 1)^2^[1+ sin^2^(2*πx*_2_)]	2	[−10, 10]	0
RotatedEllipse01	$f_{5}\left(x\right)=0.7x_{1}^{2}-6\sqrt{3}x_{1}x_{2}+13x_{2}^{2}$	2	[−10, 10]	0
RotatedEllipse01	*f*_6_(*x*)=*x*_1_^2^ − *x*_1_*x*_2_+*x*_2_^2^	2	[−10, 10]	0
Lévy	*f*_7_(*x*)= sin^2^(*πw*_*i*_)+∑_*i*=1_^*D*−1^(*w*_*i*_ − 1)^2^[1+10 sin^2^(*πw*_*i*_+1)]+(*w*_*D*_ − 1)^2^[1+ sin^2^(2*πw*_*D*_)], *w*_*i*_=1+(*x*_*i*_ − 1)/4	20/50/100	[−2, 2]	0
Sum of different powers	*f*_8_(*x*)=∑_*i*=1_^*D*^|*x*_*i*_|^*i*+1^	20/50/100	[−1, 1]	0

**Table 2:** Testing results for functions.

Function	Index	Algorithm				
		LBAS	BAS	GA	FPA	PSO
*f*_1_	Min	9.3543*E* − 14	2.0094*E* − 04	0.0018	2.9439 *E* − 04	0.0022
	Max	1.8460*E* − 08	0.1107	0.0207	0.0980	0.0431
	Med	2.4384*E* − 10	0.0317	0.0052	0.0035	0.0240
	Std	5.7173*E* − 09	0.0337	0.0070	0.0303	0.0107

*f*_2_	Min	2.8470*E* − 18	3.7867*E* − 06	3.7223*E* − 04	3.1118*E* − 05	1.0878*E* − 05
	Max	5.4943*E* − 16	4.0530*E* − 05	0.0049	0.0010	0.0790
	Med	2.2494*E* − 17	7.5555*E* − 06	4.4090*E* − 04	4.4109*E* − 04	0.0307
	Std	1.7480*E* − 16	1.4111*E* − 05	0.0014	0.0003659	0.0298

*f*_3_	Min	6.1066*E* − 13	4.9536*E* − 04	0.0048	3.5590*E* − 05	0.0032
	Max	3.6718*E* − 10	0.0102	0.0298	0.0219	0.1228
	Med	3.4965*E* − 12	0.0049	0.0068	0.0081	0.0141
	Std	1.2112*E* − 10	0.0031	0.0076	0.0070	0.0357

*f*_4_	Min	1.3498*E* − 31	1.1045*E* − 04	0.0042	1.6521*E* − 06	0.0035
	Max	1.3498*E* − 31	0.0069	0.0042	0.0021	0.0464
	Med	1.3498*E* − 31	0.0015	0.0042	3.2458*E* − 04	0.0088
	Std	0	0.0020	0	6.9832*E* − 04	0.0147

*f*_5_	Min	8.4648*E* − 40	7.1031*E* − 25	9.1805*E* − 04	3.0686*E* − 06	4.1744*E* − 05
	Max	7.1578*E* − 38	8.4409*E* − 23	9.1805*E* − 04	4.1650*E* − 04	0.0241
	Med	1.0337*E* − 38	9.4800*E* − 24	9.1805*E* − 04	6.6203*E* − 05	0.0030
	Std	2.2468*E* − 38	2.7938*E* − 23	0	1.3922E − 04	0.0087

*f*_6_	Min	1.4789*E* − 40	8.0750*E* − 25	9.5554*E* − 05	1.4530*E* − 07	5.1231*E* − 05
	Max	7.8752*E* − 39	5.8238*E* − 24	9.5554*E* − 05	5.4512*E* − 04	7.0563*E* − 05
	Med	1.8900*E* − 39	2.4073*E* − 24	9.5554*E* − 05	1.4121*E* − 05	3.5071*E* − 05
	Std	2.4521*E* − 39	1.7356*E* − 24	0	1.6684*E* − 04	2.3942*E* − 05

*f*_7(*D*=20)_	Min	1.3333*E* − 10	0.6525	0.7630	1.4159	0.2852
	Max	9.6746*E* − 10	0.8964	1.4531	4.0867	1.4176
	Med	4.9611*E* − 10	0.7412	1.1469	2.7236	0.6857
	Std	2.9712*E* − 10	0.0961	0.1920	0.7638	0.4553

*f*_7(*D*=50)_	Min	0.0181	3.7917	7.2038	11.0403	1.8572
	Max	0.1508	5.1570	8.6709	22.6117	6.0086
	Med	0.0442	4.5712	8.0477	17.7340	4.4466
	Std	0.0393	0.5305	0.5307	3.2205	1.2682

*f*_7(*D*=100)_	Min	3.0291	13.7998	23.8633	36.3632	9.4442
	Max	5.1241	17.2209	33.1076	61.4086	16.1300
	Med	3.9682	15.1314	29.6946	55.1701	11.2001
	Std	0.6243	1.1070	3.1453	9.3947	1.8171

*f*_8(*D*=20)_	Min	1.6945*E* − 11	1.9763*E* − 04	0.0024	0.0346	4.1224*E* − 05
	Max	1.8792*E* − 08	0.0011	0.0130	0.1465	7.3283*E* − 04
	Med	1.0068*E* − 09	4.1420*E* − 04	0.0053	0.0966	2.0707*E* − 04
	Std	5.8554*E* − 09	2.8560*E* − 04	0.0037	0.0377	2.0507*E* − 05

*f*_8(*D*=50)_	Min	1.3834*E* − 06	0.0016	0.0109	0.3384	4.8286*E* − 04
	Max	0.0060	0.0099	0.1248	1.1723	0.0028
	Med	5.2133*E* − 05	0.0035	0.0361	0.5372	9.4342*E* − 04
	Std	0.0018	0.0024	0.0321	0.2876	6.7667*E* − 04

*f*_8(*D*=100)_	Min	4.1213*E* − 05	8.4831*E* − 04	0.0441	0.3753	1.0003*E* − 04
	Max	0.0021	0.0096	0.1648	1.9295	0.0020
	Med	3.2448*E* − 04	0.0024	0.1085	1.3901	9.4257*E* − 04
	Std	6.1046*E* − 04	0.0029	0.0374	0.4467	6.7022*E* − 04

**Table 3:** Time domain characteristic.

Algorithm	Index					
	ITAE	*M*_*p*_	*t*_*p*_	*t*_*d*_	*t*_*s*_	*e*_*r*_
LBAS	3.6806*E* − 04	0.0773	0.0562	0.0354	0.0637	0.0002
GA	7.3866*E* − 04	0.0974	0.0753	0.0434	0.0928	0.0030
FPA	0.0022	0.0026	0.2105	0.0598	0.1199	0.0017
PSO	8.2134*E* − 04	0.1354	0.0855	0.0440	0.1145	0.0145

**Table 4:** Frequency domain characteristic.

Amplitude	Angular	Index	Algorithm			
			LBAS	GA	FPA	PSO
1	10	*∆A*_*ω*_	0	0.0030	0.0336	0.0320
		*φ*_*ω*_	0.0148	0.0274	0.0586	0.0334

5	15	*∆A*_*ω*_	0.0002	0.0108	0.0874	0.0514
		*φ*_*ω*_	0.0147	0.0268	0.0564	0.0328

10	20	*∆A*_*ω*_	0.0010	0.0270	0.1560	0.0720
		*φ*_*ω*_	0.0155	0.0282	0.0564	0.0351

15	25	*∆A*_*ω*_	0.0007	0.0453	0.2327	0.0887
		*φ*_*ω*_	0.0149	0.0273	0.0544	0.0354

20	30	*∆A*_*ω*_	0.0010	0.0715	0.3140	0.0920
		*φ*_*ω*_	0.0148	0.0281	0.0524	0.0358

25	35	*∆A*_*ω*_	0.0016	0.0676	0.3556	0.1128
		*φ*_*ω*_	0.0150	0.0291	0.0502	0.0383

**Table 5:** Ramp response characteristic.

Ideal value	Index	Algorithm			
		LBAS	GA	FPA	PSO
1	*S*	1.0005	0.9965	0.9993	0.9946
	*∆S*	0.0005	0.0035	0.0007	0.0054

200	*S*	199.6464	199.4263	200.1590	195.1578
	*∆S*	0.3536	0.5737	0.1590	4.8422

## Data Availability

The data used to support the findings of this study are available from the corresponding author upon request.
